# Acceptability of a Whatsapp Triage, Referral, and Transfer System for Obstetric Patients in Rural Liberia

**DOI:** 10.5334/aogh.4030

**Published:** 2023-05-29

**Authors:** Christopher W. Reynolds, Madison Horton, HaEun Lee, Wahdae-Mai Harmon, Joseph Sieka, Nancy Lockhart, Jody R. Lori

**Affiliations:** 1 University of Michigan Medical School, Ann Arbor, MI, US; 2 Columbia University School of Nursing, New York City, NY, US; 3 Center for Global Health Equity, University of Michigan, Ann Arbor, MI, US; 4 University of Liberia, Monrovia, Liberia; 5 University of Michigan School of Nursing, Ann Arbor, MI, US

**Keywords:** Obstetric triage, Liberia, global health, mHealth, WhatsApp, pre-hospital communication, mobile technology

## Abstract

**Background:**

Maternal mortality continues to disproportionately affect low- and middle-income countries, including Liberia. Though the relationship between obstetric triage systems and improved maternal outcomes is well documented, standardized triage protocols are lacking in rural Liberia. Mobile health interventions are a promising method to triage obstetric patients.

**Objectives:**

This study explores the acceptability of a WhatsApp Triage, Referral, and Transfer (WAT-RT) system among Liberian midwives and community health assistants.

**Methods:**

Individual interviews and focus group discussions were conducted among midwives (n = 18) and community health assistants (n = 112). Interviews were designed to understand the current referral system in rural Liberia, how a WAT-RT System can address referral limitations, and the acceptability of the WAT-RT System. Data were audio recorded, transcribed, and translated into English. Data analysis was conducted via NVivo12 with independent and cooperative techniques among multiple researchers.

**Findings:**

The current referral system is not standardized with limitations including a lack of triage protocols, transportation difficulties, and inconsistent communication of patient information, which could be addressed by a WAT-RT System. The acceptability for the WAT-RT System was high. Facilitators to implementation included utilizing a pre-existing communication and referral infrastructure, access and competency surrounding mobile phones, and increased opportunities for training and inter-provider collaboration. Barriers included disproportionate phone access between midwives and community health assistants, network reliability, and a lack of data standards. Recommendations for successful implementation included centralizing phone financing and standardizing triage protocols.

**Conclusions:**

The WAT-RT System demonstrated high acceptability among frontline health care providers in rural Liberia. Barriers to program success could be reasonably addressed with simple interventions and planning. Multiple benefits included addressing care delays for obstetric patients, promoting bidirectional provider communication, and increasing the quality of obstetric triage. Future studies should focus on piloting the WAT-RT System among this population and recruiting other key stakeholders to determine intervention feasibility.

## Background

Maternal mortality continues to exist as one of the world’s greatest examples of health care inequality [1]. Disproportionately affecting low-and-middle-income countries (LMICs) [2], maternal mortality in Sub-Saharan Africa (SSA) accounts for approximately 66% of the estimated global burden of maternal deaths [3]. Liberia, a country of approximately five million people, has one of the worst maternal mortality rates in the world, currently ranked ninth globally at 661 deaths per 100,000 live births [4]. Liberia has struggled to reduce maternal mortality, due to natural and structural forces including decades long Civil Wars, the 2014–2016 Ebola epidemic, and most recently the COVID-19 pandemic [5]. In Liberia, where more than half of the population is considered to live in abject poverty, health care resources, staff, infrastructure, and training are scarce [6].

Maternal deaths can be prevented by addressing the personal, pre-hospital, and intra-facility delays at the community and district level [4]. The “three delays” model, widely accepted in obstetric triage, conceptualizes these three steps as a framework to address various delays in care delivery for obstetric patients [7]. While interventions for obstetric triage have typically focused on patient factors and pre-hospital delays, less attention has been focused on “the third delay”—intra-facility delays affecting patients after arrival to a health institution [8,9]. In Liberia, most health systems lack a standard referral and triage protocol for obstetric emergencies. Multiple tools with varying levels of reliability have been validated across diverse settings, but none have been widely adopted by the Ministry of Health or regional care centers in Libera [10].

Mobile health, often referred to as mHealth, shows promise in better understanding and reducing delays for care seeking [11]. Many mHealth interventions across SSA have been targeted specifically towards maternal and child mortality, but with inconsistently collected metrics and varying results for patient outcomes [12]. Guidelines aimed at implementing health technologies recommend integrating mHealth solutions into existing health system functions, rather than developing standalone solutions [13]. WhatsApp, a commonly used mobile communication platform throughout much of the world, including SSA, has demonstrated usability as an mHealth intervention. Piloted in studies covering infectious disease [14], medical education and research [15,16], and preventive medicine [17], WhatsApp holds promise as a commonly used, low-cost, and secure data messaging system but has been underexplored as a potential platform to address delays in obstetric care [18]. Midwives and community health assistants (CHAs) serve as the frontline providers for obstetric care in rural Liberia. They provide basic obstetric and peripartum care and play a critical role in transferring patients to higher-care hospital facilities in emergency and complicated obstetric cases. This study assessed the acceptability of a WhatsApp Triage, Referral, and Transfer (WAT-RT) System among CHAs and midwives.

## Methods

### Study design

This qualitative study utilized in-depth individual interviews (IID) and focus group discussions (FGDs) to assess the acceptability of a WhatsApp Triage, Referral, and Transfer (WAT-RT) System. A semi-structured individual interview script was developed by members of the research team consisting of US and Liberian health professionals and qualitative research experts. Following an extensive literature review on obstetric triage protocols and pre-hospital communication tools, the interview script was piloted among Liberia health care workers to clarify questions and purge superfluous items.

The final interview script took approximately 30 minutes to complete, and consisted of five sections including current triage protocols, referral pathways, inter-provider communication, technology use, and recommendations for implementation of the WAT-RT System. Question examples included: “What makes the difference between women getting care right away and not?” and “Do you know about WhatsApp?” Acceptability was assessed by examining dynamics of current referral pathways, inter-provider communication, and technology access and competency.

### Data collection and population

During July 2021, a total of 18 individual interviews and 20 focus groups were conducted across Bong County, Liberia. Inclusion criteria for participating in the individual interviews or focus group discussions (FGDs) included: 1) currently employed as a community health assistant (CHA) or nurse/midwife serving one of the Rural health facility in the study, 2) willingness to participate in the study, 3) able to speak Kpelle or English, 4) owner of a cellphone capable of using the WhatsApp platform for secure, end-to-end encrypted communication, and 5) over the age of 18 years.

Community health assistants and midwives were recruited through three avenues: a written notice sent to RHFs by the research team, a CHA recruitment announcement by RHF staff, and word-of-mouth and cell phone communication from hospital facilities to RHFs. Individuals who met criteria of currently working and age greater than 18 years old were provided written informed consent forms and given the chance to ask questions before beginning the interview.

Focus group discussions and individual interviews were conducted in Bong County at the rural health facilities by research team members from the University of Liberia. Because the rural health facilities are staffed by only one to two midwives, individual interviews were conducted. Community health assistants for each catchment area participated as a group in the FGDs. Focus group discussions lasted approximately 45–60 minutes and used a modified version of the individual interview script which was adapted for group participation. To encourage discussion, follow up, open-ended, and probing questions, such as: “Can you tell me more about that?” were asked. All interviews and FGDs were audio recorded via a tape recorder and notes were taken on paper by research assistants. Recordings were uploaded immediately to an encrypted DropBox folder only accessible to study researchers. Hand-written notes were promptly stored in a secure lock-box at the University of Liberia.

### Analysis

Study participants were assigned a specific code used throughout analysis to guarantee anonymity. Audio-recordings were transcribed by hand into Microsoft Word and stored on an encrypted laptop. Three researchers underwent a process of thematic analysis of the qualitative data according to Kiger et al. [19] A thematic codebook was developed with six major codes: referral pathways, pre-hospital delays, facility delays, inter-provider communication, technology access and use, and recommendations (Appendix 1). The codebook and interview transcripts were exported to NVivo12, where study researchers underwent independent coding of the data following immersion in selected transcripts and the codebook. Once data were coded, investigators met collectively to confirm inter-rater agreement and categorize themed data according to findings. Researchers also differentiated responses among sub-populations by role (midwife vs. CHA), baseline inter-provider interactions, and participant distance from the main hospital. Quotes reflecting major themes were selected to include a range of participant roles and locations. To ensure data reliability, the following designs were implemented: 1) source triangulation by interviewing midwives and CHAs, 2) thematic codebook development with three team researchers using independent and cooperative techniques, and 3) consensus on final results agreed upon by all members of the research team.

### Ethics/Approval

All participants were required to undergo a process of written, informed consent before participation. The study protocol was approved by the University of Liberia and University of Michigan ethical review boards.

## Results

### Demographics

A total of 130 participants were included in the study, 18 midwives in individual interviews and 112 CHAs in 20 different FGDs. All participants were from rural Bong County, Liberia and were actively practicing as midwives and CHAs at the time of interview.

### Referral pathways

Participants described the current system of referral pathways implemented in rural Liberia to reduce morbidity due to delayed transport to hospital centers. Midwives explained that once pregnant patients present to rural health facilities, security at the facility alerts the on-call health workers. Health staff then conduct a comprehensive assessment of the patient including checking the blood pressure, temperature, physical exam, reproductive history, fetal heart rate, and determine whether the patient requires additional referral or not. One midwife stated, “We do our physical examination and checkup, like if they had previous C-section because there are some cases we can’t handle here … [if] they have huge abdomen or multi-gestation, we only open an IV line, do the blood pressure, and tell them to find a car for transport.” If the patient was stable, she would receive monitoring and care at the RHF, including malaria testing, medication for preeclampsia, and basic antibiotics. If the provider determined the patient to be unstable at the initial assessment, she would: “not leave the patient unstable …. You have to stabilize the patient before you refer them” [CHA].

Most CHAs and midwives said they would refer patients with an obstetric complication if the woman had a positive malaria test, previous cesarean-section, multiple-gestation, and/or fetal malpresentation. While the 18 midwives collectively named all components of the initial assessment, most were only able to name one or two components, and no health provider outlined systematic steps of a standardized triage protocol with a step-wise approach. There was no comprehensive list of inclusion and exclusion criteria for referral, but rather depended on individual clinician’s expertise and decision-making process.

### Pre-hospital delays

Significant pre-hospital delays persisted even after patients’ presentation to RHFs. Financial and transportation barriers were most frequently mentioned to affect obstetric patients’ ability to complete referrals. Without consistent emergency medical services, ambulance transport was rare, placing financial burdens for transport on patients through private services: “We have a lot of constraint because some people can’t finance to take a motorbike or put them in a car” [CHA]. Long distances and poor road infrastructure delayed transport. Patients traveled hours over dirt roads, often on motorbike, which became impossible during the rainy season due to flooded roads. When private cars were available, they often lacked fuel to arrive before breaking down. Without private transport, patients could solicit the hammock group, a cadre of men who carry patients via hammock to the hospital, “For some it is hard to reach the community. Sometimes they delay because of the car, and if the distance is bad, the family will call the hammock group, but this can take four or five hours. For women with complications, it is even harder to transfer” [Midwife].

Individual and interpersonal-level barriers affected timely care, including patient’s unwillingness to seek care and family member’s decision-making powers. Women were labeled as “stubborn,” “hiding” from midwives, and “feeling weak [such that] they don’t want to come” [Midwife]. One CHA described, “we have to counsel the patient because some [are] stubborn. Sometimes they say ‘I’m not going to clinic.’ So you have to talk to them and they will say ‘I want your help to carry me.’” Family members had significant influence over the women’s ability to seek care. While some prohibited patients from accessing hospitals due to the financial burden, others did not trust facilities to care for their family and unborn child:

It can cause delay, the decision of the husband at times especially to the maternal waiting home. When [the women] reach eight months, you tell them to come, but maybe the husband will say he ain’t ready yet for the person to come. And then [there is] no food so he is not prepared, so it can cause delay. [Midwife]

### Facility delays

Rural health facility and hospital facility level delays were largely caused by a lack of resources including medications, antibiotics, needles, syringes, IV tubing, and endotracheal tubes. When resources were not available, patients or families were required to buy their own supplies from local pharmacies, which were not always open or stocked: “At night … they [midwives] can send me to go buy drugs but the drugstore owner will be in bed, so it can be hard to get the medicine at night” [CHA]. Health providers could not treat a patient until supplies had been purchased. Providers worried that this hospital policy created preventable delays and morbidity in emergent cases including hemorrhage, eclampsia, or sepsis. One midwife recounted, “Even a person with a very high temperature needs to go buy [antibiotics] from the drug store and bring it. So, this will cause delay, but for those who bring it, they can be served right away” [Midwife]. Participants agreed that patients arriving in early stages of labor with already purchased supplies were the most manageable to care for.

Inconsistency in data collection and transfer infrastructure contributed to facility delays. When patients are referred, they would be sent with a referral form, completed by the RHF provider, including information such as the time of referral, patient’s condition and status, a brief history of present illness, and any therapies performed: “[This process] can make it easy when people come here. We have the community health worker, referral paper, and the condition of the patient on the paper. Right away, the patient presents at registration and that paper can help them go through the process faster” [CHA].

Upon arrival at the RHF, a registrar performed intake in a paper logbook. However, time of presentation affected how patient information was recorded. One participant explained the inconsistencies: “When working time is over from 8am–4pm, I have to take care of you. Or I could send for the midwife if I am not around. If they aren’t here, security will do the calling. If the registrar is not available, we put the information on a piece of paper until they come, and then we can register the patient” [Midwife]. Participants lamented that logbook data was inconstantly recorded, varying in completeness and specificity, even among the same patients.

Electronic medical records were nonexistent, creating challenges gathering holistic patient history such as prenatal and vaccination histories. While women had checklist-cards documenting their prenatal histories, the physical card could be lost or destroyed and the hospital did not store copies for backup. Hence, midwives and CHAs often lacked access to patients’ prenatal information. One CHA stated, “we get this information from the trained traditional midwives because they can get ahold of this information” [CHA].

### Inter-provider communication

A second aim of this study examined inter-provider communication as a key aspect of a WAT-RT System. Overall, communication between health professionals differed across communities. At minimum, providers communicated through inconsistently completed, unidirectional referral forms. At most, midwives, CHAs, and physicians met monthly to conduct Death and Complications meetings with use of formal verbal autopsies, where issues with the referral pathways could be addressed as a collective team: “There is good relationship because we have meeting every third Friday, where we can communicate. So if there is problem in their community, we tell about what to do and we will go there quickly to solve the problem and come back” [Midwife]. Most communities had an approach between these extremes, utilizing referral forms regularly and occasional phone calls for emergent cases. Participants provided examples where phones were used to expedite care for emergent patients, including one with eclampsia:

I once called my boss saying, ‘I don’t understand this case, so what is the way forward?’ So, he showed me some things to do and it came out positive. The person was not conversing, but their BP was still high. There were many transport delays, so I called other people that have been in the field for a long time, and they were able to help me through the birthing process. She gave birth and the BP continued to drop until it was a successful outcome. [Midwife]

Rural health facility workers lamented a lack of follow-up after patients were transferred to hospital facilities. Following transfer, RHFs have no method for knowing if the patient reached the hospital, their obstetric course, or final health outcomes. Midwives and CHAs found it difficult to receive feedback on opportunities for improvement without this outcomes data. Some CHAs relied on family members and trained traditional midwives to orally communicate medical and treatment information to facility providers: “If I bring any patient [to the hospital], the family is asked about what happened, and we ask the relative to stay near her to give us that information” [CHA]. Multiple pieces of medical information were important during this transfer, which was done without considering family members’ medical literacy or confirming understanding. Some worried that this practice resulted in inaccurate and incomplete information transfer.

Universally, participants spoke about colleagues with respect and professionalism. Midwives referred to CHAs as “eyewitnesses” of the communities, to relay information which allows midwives to perform their jobs more efficiently. Participants who belonged to communities with more frequent interdisciplinary contact were more likely to offer unprompted cross-specialty praise: “If something happens in the community, [CHAs] can call us right away to give us information, even taking the patient name and making sure they come to the facility. They do extremely well for us and make personal sacrifices to make sure they bring the patient here” [Midwife].

### Technology access and usage

The final aim of this study was also to assess mobile technology access and competency. There were noticeable differences between midwife and CHA participants (Tables 1a & 1b). Among CHAs, most participants reported having a phone that could send text messages (n = 72, 64.2%), but only one-third owned smartphones (n = 37, 33%). While many respondents used their phone everyday (n = 78, 69.6%), only 11 participants regularly used WhatsApp (9.8%). Compared to CHAs, midwives had higher usage of cell phones (n = 18, 100%), smartphones (n = 13, 72.2%), and WhatsApp (n = 9, 50%).

**Table 1a:** Cell phone access and usages among community health assistants (n = 112) in Bong County, Liberia.


	N	FREQUENCY

Uses phone to communicate with other health care providers	83	74.1%

Uses phone daily	78	69.6%

Personal phone that can send text messages	72	64.2%

Personal phone is a smartphone	37	33%

Uses WhatsApp	11	9.8%


Focus group discussions conducted with community health assistants in Bong County Liberia. Among 20 focus group discussions, 112 CHAs participated in groups of 3-14 participants each.

**Table 1b:** Cell phone access and usages among Midwives (n = 18) in Bong County, Liberia.


	N	FREQUENCY

Uses phone to communicate with other health care providers	18	100%

Uses phone daily	18	100%

Personal phone that can send text messages	18	100%

Personal phone is a smartphone	13	72.2%

Uses WhatsApp	9	50%


Individual interviews conducted with 18 midwives in Bong County, Liberia.

Across CHAs (n = 83) and midwives (n = 18), mobile cell phones were frequently used for inter-provider communication, including sharing patient information (n = 101, 90.2%). A CHA mentioned, “I enjoy using my phone because it can let you know what you need to do, all you need to attend to. This morning my co-workers called me and through the phone I was able to get information about some of the family problems in our community. The phone call for me is very important” [CHA]. Five midwives mentioned concerns about network reliability and connectivity, particularly in rural areas of Bong County: “The idea is good. But looking at the network system here, especially in the rural area, our concern could be the network problem” [Midwife].

### Benefits & Recommendations

CHA and midwife participants believed that a WAT-RT System could provide significant value to patients in rural Liberia, identifying facilitators and barriers to an electronic platform for communication (Table 2). Participants believed it would better prepare receiving facilities when dealing with emergency patients: “It will be ok, as soon as an emergency comes, they will be able to let us know sooner so we can prepare ahead of time if the person is bleeding. We can get ourselves set” [Midwife]. Focus group participant CHAs agreed: “I support the idea for the WhatsApp triage to be established. We would know the differences between the sick and the not sick because for the sick, they should be isolated to a specific place.”

**Table 2:** Facilitators and Barriers to WAT-RT System in rural Liberia.


FACILITATORS	BARRIERS

– Utilizes pre-existing infrastructure with referral pathways that are proven to be successful	– Financial support for consistent mobile data

– RHFs staff medical professionals accustomed to communicating with facility-level providers	– Inconsistent access to power sources for phone charging

– Desires from rural facility providers to have obstetric triage training	– Lack of standard triage protocols

– Strong interest and desire for the program among midwives and CHAs	– Lack of referral decision standards

– Involves facility providers earlier, which may reassure family who otherwise refuse transport	– Unwillingness from “stubborn” patients to have their health information shared

– Central hub to store patient information which could be widely accessible indefinitely	– No standard for what patient information is transferred or how it is collected

– Cost effective: presents no cost to patients and low-cost to health care providers	– Must be integrated to the inconsistently used rural health center referral form

– High access to smartphones among midwives	– Low access to smartphones among CHAs

– Bidirectional communication allows family to purchase supplies before arrival to the facility	– Low familiarity with WhatsApp among CHAs

– Desire from RHF to receive patient outcomes and follow-up outpatient appointments	– Inconsistent network connectivity


Participants acknowledged a desire for standardized data keeping, including staff specifically assigned to record patient registration, time to care, and clinical outcomes. They believed a bi-directional WAT-RT System could institutionalize electronic data recording and storying for simultaneous use by RHFs, hospitals, health workers, and researchers. Rural health facilities perceived benefit from receiving feedback on patients referred to those hospitals, including clinical outcomes:

The entire idea is good, because at times, we will transfer patients to the bigger hospital but then we can’t really get their feedback to know whether maybe the patient was successful to deliver by herself or maybe the patient went under C-section. Sometimes we really don’t get feedback. [Midwife]

Participants believed bi-directional information flow would facilitate tracking patient outcomes, and prepare RHFs for postpartum appointments in the weeks after delivery. Lastly, participants mentioned that such a platform would create a consultation service in which multiple health professionals could converse about complex patients, supporting the primary provider for safer care:

Another important thing about the WhatsApp triage and referral system is that, for example, all of us are CHAs right? If I have certain patient that is too much for me, I will put it in the WhatsApp group to ask a question, and all the other CHAs, chief midwives, or whoever is there will see it and answer my question quick. You don’t have to know that person first before they can answer you, but because you asked question and they have an idea on it and they are in the group, that’s why they’re answering it. It’s like you are collecting everybody from all over to put them in one room like the way we all are sitting here, that’s how this whole WhatsApp thing will be. [CHA]

Several recommendations were provided to increase successful implementation of the WAT-RT System. Participants recommended providing professional phones which would belong to RHF and hospital facilities. This would track accountability, centralize project expenses, and allow CHAs and midwives without consistent phone access to participate. To manage inconsistent access to electricity to recharge phones, they recommended solar powered battery chargers. While hospital facilities had electricity with backup generators, RHFs without consistent power could benefit from backup sources. Participants recommended county-wide training on obstetric triage and WhatsApp usage, to standardize assessment, referral, and communication of patient information to other providers.

## Discussion

This qualitative study utilized IIDs and FGDs with front-line health care providers to determine the current referral system, barriers to the referral systems, and the acceptability of a WAT-RT System for obstetric patients in rural Liberia. Our findings demonstrated high need for an improved referral system as well as high acceptability of the WAT-RT System.

Overall, study participants were unanimously excited to participate and perceived significant benefit of the WAT-RT System. Participants believed their obstetric patients would benefit from decreased pre-hospital transport and triage delays within facilities with a WAT-RT System. Such a system would directly address several of the common delays in receiving care, particularly intra-facility (Table 3). Facilitators to program implementation included utilizing pre-existing infrastructure and organizational culture, including successfully proven referral pathways, inter-provider communication, and accessible mobile phones for transmission of patient data. Barriers included a lack of standard triage protocols, lower access to smartphones and data among community health assistants, and inconsistent power and network connectivity. Recommendations to address these barriers included providing county-wide obstetric triage trainings to standardize practice and providing facility-owned phones to centralize accountability and data financing. Other potential benefits included opportunities for standardized triage training, cost-effectiveness of the intervention, and consultation platform for interdisciplinary health professionals to discuss complex cases. Additionally, WAT-RT could centralize patient data collection, storage, and dissemination, allowing for bi-directional communication, feedback, and outcomes tracking.

**Table 3:** Factors Affecting Timely Care for Obstetric Patients in Rural Liberia.


STAGE OF CARE SEEKING	FACTOR	ADDRESSABLE BY WAT-RT SYSTEM?

Pre-hospital	Transport barriers, lack of private transportation or reliable ambulance	Yes

	Financial barriers	No

	Geographic distances to facilities	No

	Patient unwillingness	No

	Family dynamics	No

Facility delays	Lack of standard triage protocols	Yes

	Lack of standard for when to refer to higher level care	Yes

	Insufficient data collection infrastructure	Yes

	Inaccurate communication of patient information	Yes

	Reliance on family to communicate health information	Yes

	Resource insufficiencies	No

	Policy of payment before treatment	No

	Desire to be cared for by female providers	No

Post-delivery care	Rural health facilities do not receive confirmation of arrival or health status of mom or baby	Yes

	No opportunities for RHF to improve care based on outcomes	Yes

	RHFs unsure when they should see mother and baby for follow-up appointment	Yes


Differences across health care role, inter-provider communication, and location were present within our sample. Specifically, midwives had greater access to mobile and smartphone technology and increased familiarity with WhatsApp compared with CHAs. This finding, along with participant recommendations, calls for providing facility-owned phones and a WhatsApp training session as the program is initiated. Inter-provider communication was strong across all locations and health professions, including using mobile phones to communicate about patient cases. However, participants with regular collaborative meetings and established feedback mechanisms were more likely to offer praise regarding their interdisciplinary colleagues. Locations differed significantly in terms of perceptions of phone network reliability: participants further from hospital facilities and the city center were more likely to mention network reliability as a potential barrier to program implementation.

This study contributes to the growing literature on mobile technology communication tools for obstetric triage and provides meaningful considerations regarding WAT-RT implementation in a low-resource setting. While limited novel interventions aimed to reduce the “third delay” and improve obstetric triage within facilities have been piloted in some LMIC settings, few have proposed interventions to encompass care delivery from the pre-hospital to facility setting [20,21,22]. A feasibility study in Ghana utilized WhatsApp as a platform for referring obstetric patients to higher care centers, with findings demonstrating usability by various health care professionals from different types of health centers, long variations in arrival time to facility for obstetric patients, and decreased arrival time when patients were accompanied by midwives [23]. Combining our findings with this pilot program suggest that a WAT-RT System, when assessed and implemented with appropriate adaptation, could provide a platform for obstetric triage in West Africa.

### Limitations

This study had several limitations. First, it is a qualitative study conducted with a geographically homogenous group of health care workers. Hence, though our sample size was large for a qualitative study, the viewpoints represented may not be widely generalizable. Second, our recruitment strategy was via convenience sampling. Though this was considered most appropriate for the qualitative methodology within a close-knit community of obstetric providers, a randomized sampling may have provided more representative data. Third, a select group of health providers: midwives and CHAs, were included in the sample. While the perceptions of these providers are most relevant, as they would serve as local champions and main implementers for the WAT-RT System, soliciting perspectives of other stakeholders including patients, physicians, hospital administration, and government officials is important. Despite these limitations, this study provides critical insights on what the current referral system is in rural Liberia, its limitations, how the WAT-RT System can mitigate these limitations, and the acceptability of the program if it were to be developed and implemented.

## Conclusions

This study examined the current referral practice in rural Liberia, the need for an improved referral platform and program, and the acceptability of a WhatsApp Triage, Referral, and Transfer System for obstetric patients in rural Liberia. Many facilitators, including utilizing pre-existing referral pathways, technology infrastructure, and inter-provider communication could enable program implementation, with participants listing significant benefits including decreased patient care delivery times, institutionalized and standardized data collection and storage, and opportunities for bi-directional feedback and health provider improvement. Barriers to implementation included network reliability, smartphone access, and lack of standard triage and referral protocols; all of which could be addressed with simple and cost-effective interventions before program implementation. Future studies should focus on the perceptions of patients and other health care providers, pilot testing of the WAT-RT System, examining its cost effectiveness, and measuring improvements in patient referral and health outcomes.

## Additional File

The additional file for this article can be found as follows:

10.5334/aogh.4030.s1 Appendix 1. WAT-RT NVIVO Codebook.
