# Supplementary material for: Acceptability of a Whatsapp Triage, Referral, and Transfer System for Obstetric Patients in Rural Liberia
Source: Ann Glob Health. 2023 May 29;89(1):34. doi: 10.5334/aogh.4030 (PMC10237243; doi:10.5334/aogh.4030)
Supplement: Appendix 1. — WAT-RT NVIVO Codebook. [file agh-89-1-4030-s1.pdf]

WAT-RT Codebook

Codes

Name	Description
Referral pathways	
Accessibility to health facilities	
Ambulance services	
Financial difficulty	
Other transport issues	
Road Conditions	
Pre-hospital delays	
Decision to attend health facilities	
Familial factors	
Cultural beliefs leading to delay	
Facility delays	
Delivery processes at health facilities	

Name	Description
Blood transfusion processes	
Triage of pregnant women	
Quality of health facility	
Health facilities basic resources Water, electricity and food	
Health facilities drugs and supply	
Health facility maintenance	
Patients record	
Inter-provider communication	
Technology access and use	
Smart phone availability	
Challenges with phone network	
Existing knowledge of WhatsApp App	
Challenges using WhatsApp	
Perceptions about WhatsApp triage implementation	
Recommendations	
